# Artificial Intelligence in Laryngeal Endoscopy: Systematic Review and Meta-Analysis

**DOI:** 10.3390/jcm11102752

**Published:** 2022-05-12

**Authors:** Michał Żurek, Kamil Jasak, Kazimierz Niemczyk, Anna Rzepakowska

**Affiliations:** 1 Department of Otorhinolaryngology Head and Neck Surgery, Medical University of Warsaw, 1a Banacha Str., 02-097 Warsaw, Poland; kniemczyk@wum.edu.pl (K.N.); arzepakowska@wum.edu.pl (A.R.); 2 Doctoral School, Medical University of Warsaw, 61 Żwirki i Wigury Str., 02-091 Warsaw, Poland; 3 Students Scientific Research Group, Department of Otorhinolaryngology Head and Neck Surgery, Medical University of Warsaw, 1a Banacha Str., 02-097 Warsaw, Poland; s071767@student.wum.edu.pl

**Keywords:** artificial intelligence, larynx, lesion, laryngoscopy, accuracy, specificity, sensitivity

## Abstract

Background: Early diagnosis of laryngeal lesions is necessary to begin treatment of patients as soon as possible to preserve optimal organ functions. Imaging examinations are often aided by artificial intelligence (AI) to improve quality and facilitate appropriate diagnosis. The aim of this study is to investigate diagnostic utility of AI in laryngeal endoscopy. Methods: Five databases were searched for studies implementing artificial intelligence (AI) enhanced models assessing images of laryngeal lesions taken during laryngeal endoscopy. Outcomes were analyzed in terms of accuracy, sensitivity, and specificity. Results: All 11 studies included presented an overall low risk of bias. The overall accuracy of AI models was very high (from 0.806 to 0.997). The accuracy was significantly higher in studies using a larger database. The pooled sensitivity and specificity for identification of healthy laryngeal tissue were 0.91 and 0.97, respectively. The same values for differentiation between benign and malignant lesions were 0.91 and 0.94, respectively. The comparison of the effectiveness of AI models assessing narrow band imaging and white light endoscopy images revealed no statistically significant differences (*p* = 0.409 and 0.914). Conclusion: In assessing images of laryngeal lesions, AI demonstrates extraordinarily high accuracy, sensitivity, and specificity.

## 1. Introduction

The spectrum of laryngeal pathologies is very wide, and every level of the larynx may be involved in neoplastic, pre-neoplastic or non-neoplastic processes, although the majority of changes are localized in the glottic part. Prior to laryngeal cancer, cellular changes begin with epithelial hyperplasia, then develop into dysplasia, squamous cell carcinoma in situ and eventually into invasive cancer [1,2]. Potentially, 6% to 22% of premalignant lesions will develop into malignancies, and the transformation rate depends on the severity of the precancerous lesions [2]. The aim of modern diagnostics is the proper assessment of lesions in the larynx with the lowest possible invasiveness of examination. First, it is necessary to distinguish malignant and potentially malignant lesions from benign ones. The benign vocal fold lesions classification includes nodules, polyps, cysts, fibrous masses, pseudocysts, and non-specific lesions [3].

It is crucial to perform prompt diagnosis and preoperative assessment in order to provide adequate and minimally invasive treatment to preserve organ functions [2]. Especially in the case of laryngeal cancer and its precursor lesions, the treatment process has a great influence on everyday basic functions such as breathing, swallowing, and voice production. The current approach to laryngeal cancer places great importance on preventing total laryngectomy whenever possible in order to maintain the best quality of life [1,4,5,6].

Many tools are used in the diagnosis of laryngeal lesions at different stages of advancement, including indirect and direct laryngoscopy, ultrasound, computer tomography, and magnetic resonance imaging [7]. Each of the methods has its advantages and limitations, which affects their usefulness. The diagnosis of laryngeal lesions begins primarily with indirect laryngoscopy, preferably with the endoscopy equipment [8]. The speed, ease of performance, low cost, and high efficiency of endoscopy have made it a key diagnostic tool. Various facilities have been introduced to improve the sensitivity and specificity of this examination. The original white light endoscopy (WLE) imaging has certain limitations: it provides poor quality images and with a lack of clinical experience premalignant or malignant lesions can be overlooked. WLE is not so precise in distinguishing mucosal differences, especially when assessing dysplastic and cancerous lesions at initial stages [6]. Because of certain limitations of classical WLE, some image-enhanced endoscopy techniques such as autofluorescence, contact endoscopy, and narrow band imaging (NBI) have been developed. Currently, there is an increase in the usage of these enhanced endoscopy techniques observed in everyday clinical practice, especially among patients with laryngeal pathologies [9]. In particular, the NBI technique has been shown to be more accurate in diagnosing laryngeal dysplasia compared to WLE alone [10,11]. It should be emphasized that directed biopsy and histopathology remains the gold standard for the final diagnosis of laryngeal lesions. However, biopsy is a mentally and physically demanding procedure for the patient and may cause vocal fold or laryngeal dysfunction [5,12]; therefore, less invasive diagnostic methods with a sensitivity and specificity close to histopathology results are being sought.

One of the crucial problems related to introducing a new diagnostic tool remains the learning process. The relationship between efficiency and experience is not a linear dependence. The learning speed changes depending on the level of the examined person [13]. In order to avoid limitations in access to recent diagnostic methods due to young doctors’ lack of experience, much software equipment supporting the assessment of lesions is currently being implemented. One such tool is artificial intelligence (AI), which uses computer programs to understand the capabilities of the human mind in order to imitate our problem-solving and decision-making. Since its beginning in the fifties, AI has evolved dramatically. Currently, AI may precipitate diagnosis, improve its accuracy, and have a beneficial impact on efficiency in clinical practice. The fact that some subclasses of AI allow machines to learn how to use gathered information and make decisions independently is very promising. AI can analyze an input image to recognize patterns and create specific filters in order to compute the final outcome [14]. Introducing AI into the diagnostic process in the case of medical imaging is thought to contribute to better precision, replicability, and efficiency in making diagnoses. In 2017, Arterys became the first U.S. Food and Drug Administration approved clinical application in healthcare, based on cloud-storage data [14]. CardioAI was the first Arterys product, used in the analysis of magnetic resonance heart images. Since then, the application has also been developed to analyze liver and lung imaging, chest X-ray and bone X-ray images, and head CT images without contrast. Applications based on AI are also widely used in gastroenterology.

The advantages of AI-enhanced systems have been proven many times, an example being the study of Repici et al., where a 14% increase in the adenoma detection rate was noted using an AI system [15].

The purpose of this meta-analysis is to evaluate the efficacy and clinical utility of AI in the assessment of laryngeal lesions based on laryngoscopy imaging studies. This objective will be achieved by analyzing the ability of AI to evaluate selected laryngeal lesions based on accuracy, sensitivity, and specificity.

## 2. Materials and Methods

### 2.1. Search Methods, Types of Studies, and Participants

A systematic review of the literature was undertaken to investigate the diagnostic utility of AI in laryngeal endoscopy. For the purpose of this investigation, AI was defined as expert computer systems created for predicting or classifying and based on input data. To report the results as recommended, PRISMA guidelines [16] were followed. The PICO framework model [17] was used to describe the search strategy (Table 1). The search was conducted through five publication databases (PubMed, Embase, Cochrane, Scopus, and Web of Science) by two independent scientists (MŻ and KJ), After research of databases two additional articles were retrieved. Search strategies used in the systematic review are presented in Supplementary Materials (Table S1). The words presented in Table S1 were used to find all articles with the searched topic, from which the corresponding MeSH terms were generated so that the risk of missing key studies was minimized. Publications available until 15 October 2021 were included.

All patients who underwent laryngeal endoscopic examination with a consecutive histopathological diagnosis were included in the study. Randomized controlled trials as well as retrospective and prospective cross-sectional studies, including case-control and cohort type accuracy studies, were subsumed. Animal or in vitro studies, publications not written in English, case reports, reviews or systematic literature reviews, editorials and opinion pieces, meta-analysis, and conference abstracts were excluded.

### 2.2. Index Tests and Target Conditions

Studies that examined the sensitivity, specificity, or accuracy of AI classifying laryngeal lesions based on endoscopic images were eligible. The reference standard was based on histopathological diagnoses.

### 2.3. Data Collection and Analysis/Selection Process

Study selection was divided into three phases. The first phase was the removing of duplicated results in EndNote 20 software (Clarivate Analytics, Philadelphia, PA, USA). The phase following this was screening and filtering titles and abstracts of scientific papers against inclusion and exclusion criteria. The first and second phases were realized by two reviewers (MŻ and KJ). During the third phase, the independent reviewer (AR) evaluated the full-text manuscripts for eligibility, noting the reasons for exclusions. Any inconsistencies between the reviewers were settled through conversation, until agreement was reached. A PRISMA flowchart [16] summarizing the results of data collection and analysis was created. The review protocol was registered with the International Prospective Register of Systematic Reviews (PROSPERO, CRD42021282843).

### 2.4. Risk of Bias Assessment

The quality of the studies was independently evaluated by three reviewers (MŻ, AR, KJ) using a quality assessment tool for diagnostic accuracy studies (QUADAS-2) [18]. The QUADAS-2 tool is divided into four primary domains: patient selection, index test, reference standard, and flow of patients through the study and timing of the index tests and reference standard (flow and timing). According to the authors’ recommendations of QUADAS, questions should be review-specific tailored. Due to the specific nature of the assessed studies, the domain “Patient selection” was replaced by “Materials selection.” Furthermore, additional questions were included in each domain in the QUADAS tool, and some original questions were omitted. The tailored QUADAS tool is presented in a Supplementary Materials (Table S2). Based on the results of bias assessment, clustered bar graphs were prepared.

### 2.5. Statistical Analysis and Data Synthesis

The aim of the study was to assess the clinical usefulness of AI in the laryngeal endoscopy; therefore ,the study focused on four main aspects:

(1) Analysis of the overall accuracy of AI in assessing laryngeal lesions;
(2) The ability of AI to identify healthy tissue;
(3) The ability of AI to differentiate benign lesions from potentially malignant and malignant ones;
(4) Analysis of diagnostic performance of AI using NBI and WLE images.

Before proceeding to the comparative analysis of the selected studies, it was necessary to standardize the terminology of laryngeal lesions across the studies. Most of the authors used the classifications heathy tissue and benign, precancerous, and malignant lesions, although in some papers clinical terms for changes were applied. In the research, cysts, nodules, polyps, Reinke’s edema, webs, sulcus vocalis, and laryngitis were included under benign lesions. Keratosis, leukoplakia, mild and severe dysplasia, and papillomatosis were considered precancerous. The same inhomogeneity was revealed for the vascular-pattern description of the involved laryngeal mucosa in NBI endoscopy. For consistency, it was decided to transform the nomenclature of vascularization in accordance with the most widespread classification, that of Ni [19]. Raw data were extracted from each study involved in the form of a 2 × 2 table, including the numbers of true positives (TP), false positives (FP), true negatives (TN), and false negatives (FN). A summary of the data collected and the terminology used are presented in Supplementary Materials (Tables S3 and S5).

A meta-analysis of the diagnostic accuracy of the raw data was conducted using R “meta” package version 5.0-1, “metafor” package version 3.0-2, and “nsROC” package version 1.1 (R version 4.0.2, R Foundation for Statistical Computing, Vienna, Austria). The forest plots and receiver operating characteristic (ROC) curves were performed to depict the relationship between individual and summarized values of specificity and sensitivity. Τ2 and I2 statistics were used to evaluate the studies’ heterogeneity. To assess the heterogeneity between subgroups, the test for subgroup differences was used. Sensitivity and specificity analyses using the random-effects model were conducted for both analyzed fields. Statistics with a *p*-value under 0.05 were considered significant.

## 3. Results

### 3.1. Results of the Search

**Figure 1:**
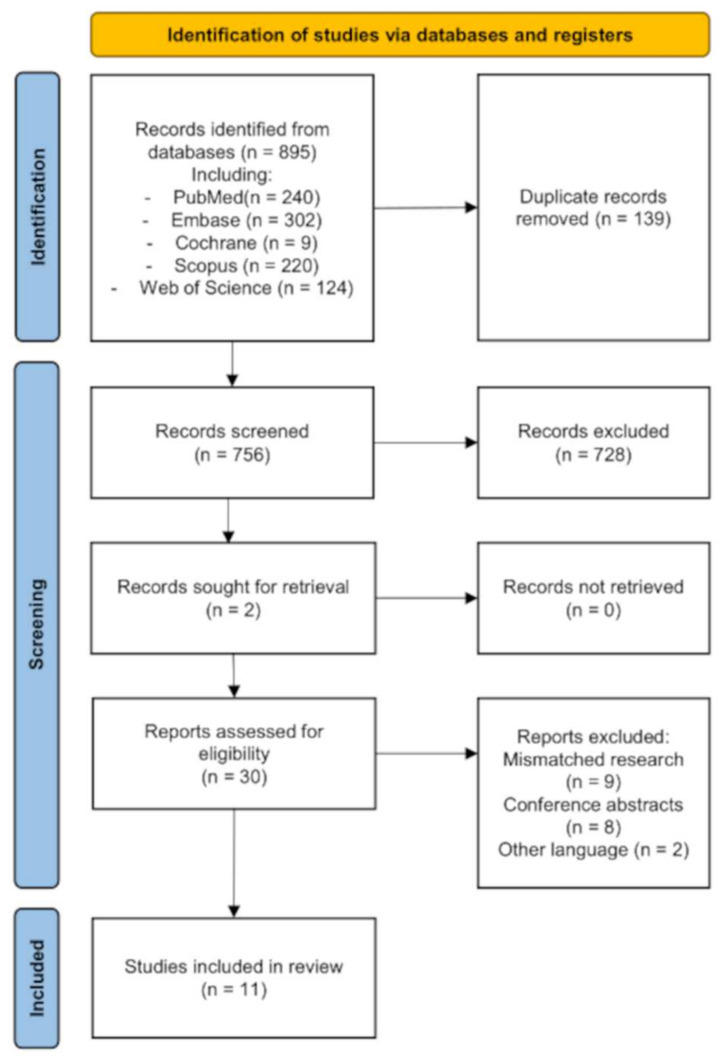
Flow diagram of the systematic review search.

Based on the literature search, a total of 895 publications were identified. After removing 139 duplicate records, 756 publications remained, which were screened by title and abstract. This led to the exclusion of 728 publications. The confrontation of the results of the literature review with another researcher resulted in retrieval of two additional records. Thus, there were 30 publications included for full-text assessment. Nineteen publications were excluded thereafter. The systematic review included 11 papers in total evaluating the diagnostic accuracy of AI in laryngeal endoscopy [20,21,22,23,24,25,26,27,28,29,30], as shown in the PRISMA flow diagram (Figure 1) and summarized in Supplementary Materials Table S3.

All the included studies were retrospective studies and used AI to assess images of laryngeal lesions. All neural networks assessed the character of the lesions on the basis of vascular patterns, shape, and/or color. Six of them evaluated endoscopic images in white light and five using the NBI method. The total number of images used in an individual study varied widely, from 120 to 24,667. Additionally, the pre-processing methods used in the included studies varied. In seven studies, images of the entire vocal folds were used, while in four studies only selected fragments of the images were evaluated. The methodology varied from the manual selection of images and their classification to complex informatic methods allowing for the extraction of specific features of the images. Seven of the studies used a pre-trained convolutional neural network (CNN) to classify the lesions; others used a support-vector machine (SVM), k-nearest neighbors (KNN), or random forest (RF) algorithms. The analysis evaluated AI’s overall diagnostic accuracy in laryngeal endoscopic procedures [20,21,22,23,24,25,26,27,28,29,30]. Concerning the different objectives of the included studies, sub-groups analyses were also performed to verify the utility of AI in clinically specific diagnostic problems:

- Identification of healthy laryngeal tissue, including seven studies [22,23,26,27,28,29,30];
- Differentiation between benign and malignant laryngeal lesions, including six studies [23,24,25,26,27,30];
- Comparison of the AI accuracy of white light endoscopy (three studies) [23,27,30] or the NBI method (three studies) [24,25,26].

### 3.2. Risk of Bias Assessment

**Figure 2:**
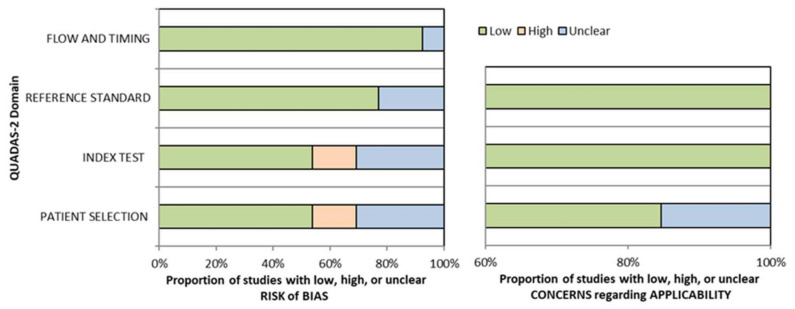
QUADAS-2 assessment of bias and applicability.

The results of the QUADAS-2 bias and applicability evaluation are summarized in Figure 2, whereas Table S4 (Supplementary Materials) lists the specific bias scores for each of the seven categories for all research included. In numerous included studies, the QUADAS-2 assessment revealed a low risk of bias.

Bias in patient selection was low in seven, unclear in two, and high in two studies. The material selection bias was difficult to assess because of specific nature of the research. The selection process of patients was not always clear and it was considered that the evaluation of the fragments of images may contribute to the reduced credibility of the research materials, which at the same time increases the risk of bias in the domain. The risk of bias in the index test was high in one study and unclear in three studies and the risk of bias in the reference standard was only unclear in one study. The reason for this result was the lack of an appropriate presentation of the results. In every study, the flow and timing risk of bias were low. Moreover, considerable variation in terminology and pre-processing methods can lead to heterogeneity in all modalities. The risks of bias in most domains were high and unclear in two studies [20,28]. The patient selection process and results in these studies have not been adequately described. In four studies [20,24,26,28], only fragments of images from patients with laryngeal carcinomas were used, which limits the randomness of the research group; therefore, the bias in patient selection was considered unclear or high. The results in two studies [20,21] were not adequately presented, which limited their usefulness in this publication.

### 3.3. Diagnostic Accuracy of AI in Assessment of Laryngeal Lesions

The first part of the analysis includes the assessment of the accuracy of all included studies. Due to the variety of research, in particular the objectives and number of research groups, the classic forest plot and ROC analysis is not recommended. The aim of this section is to indicate the potential of neural networks and their dependence on the number of images used. The accuracy of AI in assessment of laryngeal lesions differs between 0.806 to 0.997. Such high accuracy shows how valuable it is to introduce AI into everyday clinical work, regardless of the type of laryngeal lesion assessed.

**Figure 3:**
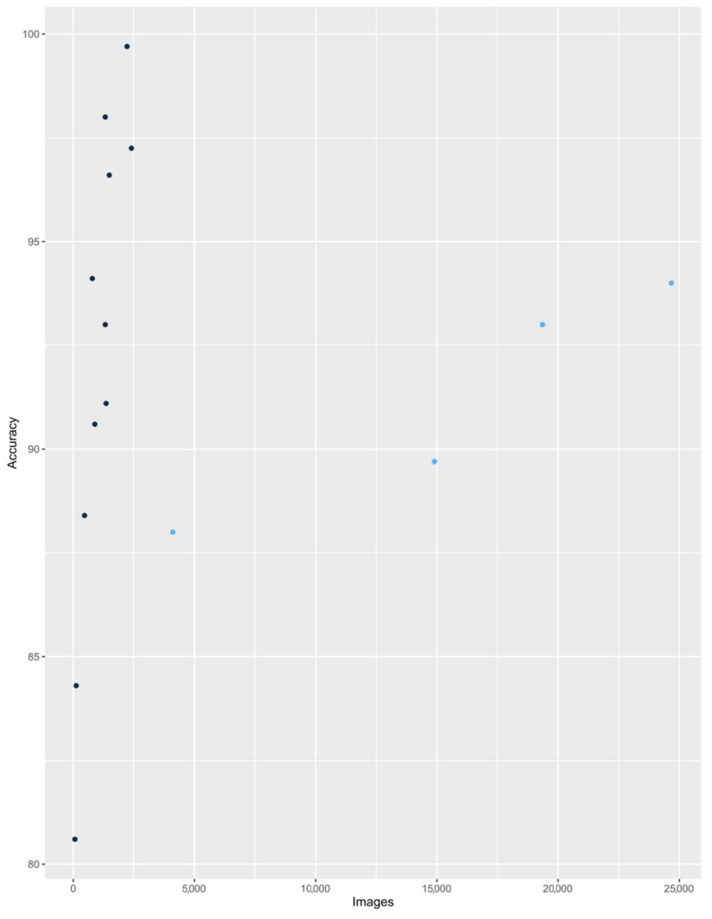
Dot plot of the accuracy of included studies (there are more dots than studies because some research analyzed more than one classification of laryngeal lesions). The dark blue points represent the group of studies for which the linear regression equation was calculated. The remaining studies are marked with light blue points.

Figure 3 shows the relationship between accuracy and the number of images per study. This figure allows one to distinguish and compare the results of two types of research, those with low and high amounts of analyzed images. In the first group of studies, a relatively small number of images beneath < 2500 were analyzed with AI and a wide range of AI accuracy was obtained, from 0.806 to 0.997 [20,21,24,25,26,28,29]. There was also an observed tendency of increasing accuracy with the quantity of applied pictures; however, it must be also stressed that in each of these studies advanced and different pre-processing methods for images were applied, including Gaussian smoothing, the investigation of texture-based global descriptors, the calculation of first-order statistics, specular reflection removal, and region of interest (ROI) detection. A linear regression curve was determined for the first group. Its formula is as follows:

ŷ = 83.67 + 0.0071 ∙ x,(1)

where y is accuracy and x is the number of images.

The assessment of the model fit is good: R^2^ = 0.7997; *p*-value = 0.0003.

For the second group, with a quantity of images exceeding 2500, an evident trend of increasing accuracy with the number of images included was recognized, from 0.88 to 0.94 [22,23,27,30]. This tendency cannot yet be confirmed statistically, due to only four studies on such scale having been performed so far, but it is worth noting that for those studies using a large number of analyzed images, the pre-processing methods were very simple compared to the first group and included only choosing images and the detection of ROI. This comparison identifies two directions for future research, in which there is an awareness of the limitations of data preparation, which should be unified and verified so as not to influence the accuracy score.

The construction of a linear regression model for all studies would not be valid due to excessive differences in their methodology.

### 3.4. Diagnostic Sensitivity and Specificity for Identification of Normal Tissue

**Figure 4:**
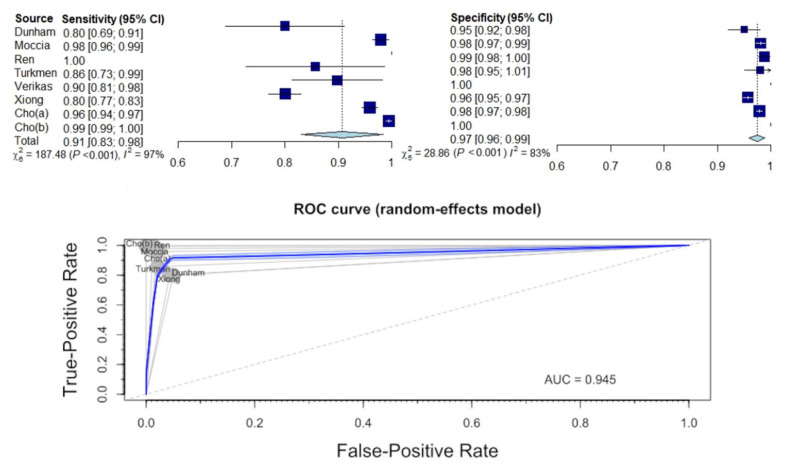
Forest plot and ROC curve illustrating the diagnostic performance of AI identifying healthy laryngeal tissue.

The diagnostic performance of AI in the identification of healthy laryngeal tissue during endoscopy is presented in Supplementary Materials (Table S5) and in Figure 4. The estimated mean sensitivity and specificity of the diagnosis of healthy tissue were 0.91 (95% CI: 0.81–1.00) and 0.97 (95% CI: 0.96–0.99), respectively. The area under the ROC curve (AUC) was 0.945.

The between-study heterogeneity variance was estimated for pooled sensitivity and specificity analysis and revealed a substantial difference between studies (sensitivity: τ^2^ = 0.0075 (95% CI: 0.0024–0.476), I^2^ = 97.3% (95% CI: 95.8–98.2%); specificity: τ^2^ = 0.0001 (95% CI: 0.0001–0.0012), I^2^ = 82.7% (95% CI: 63.4–91.8%); *p*-value for both analyses < 0.0001).

### 3.5. Diagnostic Sensitivity and Specificity for Distinguishing between Benign and Malignant Lesions

**Figure 5:**
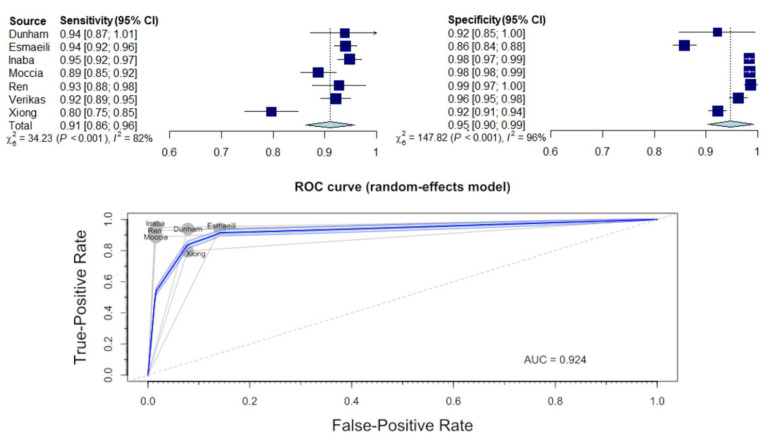
Forest plot and ROC curve illustrating the diagnostic performance of AI distinguishing benign and malignant laryngeal lesions.

The next stage of the analysis concerned the assessment of the effectiveness of AI in distinguishing benign from malignant lesions in endoscopic examinations of the larynx. The diagnostic performance is presented in Supplementary Materials (Table S5) and in Figure 5. The estimated mean sensitivity and specificity of the differential diagnosis between benign and malignant lesions was 0.91 (95% CI: 0.85–0.97) and 0.94 (95% CI: 0.89–1.00), respectively. The area under the ROC curve (AUC) was 0.924.

The pooled analysis also revealed a significant variation between studies (sensitivity: τ^2^ = 0.0027 (95% CI: 0.0008–0.0196), I^2^ = 85.4% (95% CI: 70.1–92.8%); specificity: τ^2^ = 0.0026 (95% CI: 0.0009–0.0159), I^2^ = 96.6% (95% CI: 94.6–97.9%); *p*-value for both analyses < 0.0001).

### 3.6. Comparison of Diagnostics Using WL and NBI

**Figure 6:**
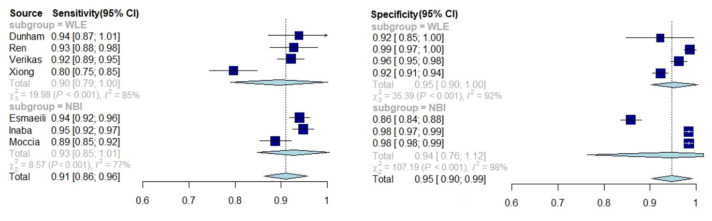
Forest plot illustrating the differences in diagnostic performance of AI using WLE and NBI.

The last part of the analysis concerns the comparison of the results depending on the whether the endoscopic method performed was WLE or NBI. This part of the analysis concerns the studies differentiating benign and malignant lesions in the larynx. The sensitivity of AI was higher for NBI (0.93, 95% CI: 0.85–1.01) than for WLE (0.89, 95% CI: 0.69–1.08). In turn, for specificity, the results were very similar: 0.94 (95% CI: 0.76–1.12) for NBI and 0.95 (95% CI: 0.85–1.04) for WLE (Figure 6). The test for subgroup differences suggests that there is no statistically significant subgroup effect (*p* = 0.409 for sensitivity and *p* = 0.914 for specificity).

## 4. Discussion

### 4.1. Main Findings

AI shows extremely high accuracy, sensitivity, and specificity in assessing images of laryngeal lesions. The accuracy of the studies cited differs between 0.806 and 0.997. Such high values indicate the great utility of AI in laryngology and provide potential opportunities to introduce AI into diagnostic standards. The regression model of accuracy of seven included studies shows a statistically significant trend between the accuracy of AI diagnoses and the number of images (*p* = 0.0003). This means that the key element to improve the quality of AI models in the assessment of laryngeal lesions is the increase in the number of images used, while maintaining high-quality pre-processing method.

In the second part, the ability of AI to identify healthy tissue was assessed. The pooled sensitivity and specificity were 0.91 and 0.97, respectively, which indicates an exceptionally high efficiency. Depending on the study, healthy tissue was differentiated from malignant lesions, such as cancer or severe dysplasia [26,27,29,30], but also from benign lesions, such as nodules, polyps, Reinke’s edemas, granulomas, or vocal fold palsies [22,23,28]. Although it is problematic to indicate the clinical usefulness of AI on this basis, the results indicate its enormous potential, and it may help young doctors learn the correct diagnosis of laryngeal lesions and, in particular, how to differentiate benign from malignant lesions.

The subsequent part evaluated the most crucial step in the diagnostics of laryngeal lesions, i.e., the differentiation of benign and malignant lesions. AI also performed very well—pooled sensitivity was 0.91 and pooled specificity was 0.94. Particularly high specificity indicates the ability of AI to discriminate patients with benign lesions from those with malignancies. These results confirm the high utility of AI in clinical practice.

The modern method of endoscopy, NBI, allows the enhanced visualization of vascular patterns and identification of neoangiogenesis accompanying carcinogenesis, which facilitates the differentiation of malignant lesions compared with WLE in clinical practice [2,6]. The results of AI accuracy for both methods were also confronted, and the results for AI assessment were comparable regardless the technology used, which is the opposite of the accuracy obtained for ENT specialists’ evaluations. The sensitivity and specificity of AI for both methods were 0.89 and 0.95 (for WLE) and 0.93 and 0.94 (for NBI), respectively. The analysis of subgroup differences shows that for AI, there are no statistically significant differences in the accuracy of differentiating benign and malignant lesions in the WLE and NBI (*p* = 0.409–0.914).

### 4.2. Association with Other Studies

The application of AI in endoscopic evaluation is currently the subject of intense research, especially in digestive track endoscopy. The main task is to enhance its performance and resolve limitations related to experience and uncertainty, and therefore implement it in modern instrument systems for the automatic detection of pathologies. The topic of laryngeal endoscopy is still at its initial stage; however, a significant increase in research has been observed in the last two years and the subject will certainly be intensively explored. At this early stage it is recommended to evaluate the essential strategies of analysis and indicate the importance of consistent data collection, the homogeneity of nomenclature, and comparable amounts of images and other technical aspects related to image processing.

For clinical reasons, the part of our meta-analysis focusing on distinguishing malignant and benign lesions seems to be the most crucial. The technical improvement of endoscopic images and their widespread adoption in the previous decade has allowed more efficient preoperative diagnosis and therefore more accurate treatment strategies for patients. Many studies assessing the effectiveness of this so-called optical biopsy in detecting malignant lesions have been performed. In the work of Davaris et al. [31], three experienced otorhinolaryngologists assessed endoscopic WLE and NBI images of laryngeal lesions, achieving a sensitivity of 0.77 (95% CI: 0.688–0.853) and 0.933 (95% CI: 0.878–0.988) and specificity of 0.973 (95% CI: 0.956–0.991) and 0.973 (95% CI: 0.956–0.991), respectively. The reference standard was histopathologic examination. In the early meta-analysis of Zhou et al. from 2018 [32] summarizing eight studies in the field of laryngeal lesions, the sensitivity and specificity in the diagnosis of malignant lesions in NBI was 0.91 (95% CI: 0.885–0.931) and 0.915 (95% CI: 0.893–0.934), respectively. Later studies supported only the evidence with the values of sensitivity and specificity ranging from 0.84 to 0.985 and from 0.889 to, 0.985, respectively [33,34,35,36,37]. It must be emphasized that in that research, the parameters of diagnostic accuracy were obtained based on evaluation by at least two specialists experienced with the method.

According to the results presented here, the effectiveness of neural networks in the diagnosis of malignant changes does not differ from the assessments of professionals. The sensitivity and specificity were 0.91 (95% CI: 0.85–0.97) and 0.94 (95% CI: 0.89–1.00), respectively. The sensitivity was relatively higher—0.93 (95% CI: 0.85–1.01)—when only the studies with the use of NBI light were assessed, which is consistent with the results of the studies cited above.

The results of the meta-analysis indicate that AI is a valuable tool for the assessment of laryngeal lesions and that the effectiveness of neural networks does not differ from the assessments of professionals. It would be particularly valuable to introduce AI in facilities that do not use NBI, because the sensitivity of the network assessments in white light (0.89) was higher than that of professionals (0.77), and the specificity was at a similar level (0.95 and 0.973, respectively).

However, it should be noted that one of the main goals of introducing AI tools in medicine is to support the work of young and inexperienced doctors. In the work of Nogués-Sabaté et al. [38], the effectiveness of the diagnosis of malignant lesions was compared using WLE and NBI images between experienced specialists and medical students. Interobserver agreement among professionals was assessed both for WLE and NBI images as substantial (κ = 0.63 and 0.68, respectively), and for trainees as moderate (κ = 0.48 and 0.55). These results confirm the need to introduce additional diagnostic tools, especially for physicians with little experience.

### 4.3. Limitations

The inaccuracies in the results of the meta-analysis with the data provided in subchapter 4.2 should be pointed out. There are some discrepancies in clinical and pathological nomenclature of laryngeal lesions. As we considered the histopathological examination as a reference standard, we accepted the classification of benign, premalignant (dysplastic), or malignant lesion as the most reasonable.

The other limitation of the meta-analysis is the considerable heterogeneity of the methodology, especially in terms of pre-processing of the images and the number of patients and images used. The most limited number of images used to assess laryngeal lesions was 120 [21] and the largest was 24,667 [27]. The pre-processing and methodology of the study is clearly related to the size of the study. A tendency was observed that the smaller the research sample, the more complicated the pre-processing. In the smallest study of Barbalata et al. [21], the preparation of images for evaluation by AI was proceeded by many steps related to the graphic processing of images, including specular reflection removal, ROI detection, blood vessel extraction, and the determination of vessel size. In contrast, the largest study, Ren et al. [27], did not process the images at all, but only manually removed duplicates and low-quality images. It should also be noted that when the studies with the same classification of laryngeal lesions (e.g., differentiation of benign and malignant lesions) are compared, the sensitivity and specificity of AI classification is at a similar level. This means that a complicated pre-processing method on a limited number of images gives similar results to using a large image database. In the study by Esmaeli et al. [24], 1485 images were used, and the AI distinguished benign and malignant lesions with a sensitivity and specificity of 0.94 and 0.86, respectively. In contrast, in the study by Ren et al. [27] (24,667 images), the same classification achieved sensitivity and specificity of 0.93 and 0.99, respectively.

The study by Arahujo et al. [20] used the first publicly available database of images of laryngeal lesions provided by Moccia et al. [26]. The Moccia et al. study [26] obtained an accuracy of 0.93, while the same classification using a different methodology in the study by Arahujo et al. [20] obtained an accuracy of 0.98. A similar situation applies to the works of Cho et al. [22,29]. The objectives of the research in both studies are different, as well as the period of material collection (in the first study 2013–2020, in the second 2010–2016), but it should be assumed that some of the images used for the research were common in both papers and the results differed significantly. Based on the above arguments, it should be concluded that the use of advanced methods of graphical image analysis on very large datasets will allow one to obtain better results and increase the clinical utility of AI models. This points to the need for large image databases and closer cooperation between the medical and IT centers.

The standard in meta-analysis is the assessment of the certainty of the evidence for outcomes. The limitation of this systematic review is the inability to use standard tools (like GRADE) to assess the certainty of the evidence [39], as there are still no standardized AI models, and each of the studies cited above used their own models. Although the results show the great advantages and potential of AI, it is still not possible to recommend one specific tool for assessing laryngeal changes on this basis. As authors, we draw attention to the need for the creation of publicly available databases of images of laryngeal lesions and the development of most accurate neural network model for laryngeal endoscopy on this basis. The development of research on the clinical application of neural networks in this direction will allow for a comprehensive evaluation and will speed up the identification of the most reliable tool in the diagnosis of laryngeal pathologies.

## 5. Conclusions

In assessing images of laryngeal lesions, AI demonstrates extraordinarily high accuracy, sensitivity, and specificity. Such high values indicate the significant utility of AI and offer an enhanced diagnostic tool in laryngology. The performance of AI diagnoses increases in efficacy with the size of the image database used for learning and testing, and with the number of pre-processing steps involving extracting specific features of the images. The best way to increase the quality and utility of AI in diagnosis is to develop standards for evaluating images and to strengthen multi-center cooperation by sharing a database of images of laryngeal lesions, which will allow the building of AI models with the best performance, based on a vast amount of images for learning and testing.

## Figures and Tables

**Table 1:** Population, Intervention, Comparison, Outcome (PICO).

PICOS Framework	
Population	Patients (without any age limit) who underwent laryngeal endoscopic examination
Intervention	Evaluation of endoscopy images by AI
Comparison	Histopathology or histopathology with specialist assessment
Outcome	Classification of laryngeal lesions

## Data Availability

Not applicable.

## Supplementary Materials

The following supporting information can be downloaded at: https://www.mdpi.com/article/10.3390/jcm11102752/s1. Supplementary Table S1: Literature search strategy; Supplementary Table S2: The tailored QUADAS questions; Supplementary Table S3: Summary table of the studies included in the meta-analysis; Supplementary Table S4: The results of the QUADAS-2 bias and applicability evaluation; Supplementary Table S5: Raw data of the included studies.

## References

[1] Hrelec C. (2021). Management of Laryngeal Dysplasia and Early Invasive Cancer. Curr. Treat. Options Oncol..

[2] Mannelli G., Cecconi L., Gallo O. (2016). Laryngeal preneoplastic lesions and cancer: Challenging diagnosis. Qualitative literature review and meta-analysis. Crit. Rev. Oncol. Hematol..

[3] Naunheim M.R., Carroll T.L. (2017). Benign vocal fold lesions: Update on nomenclature, cause, diagnosis, and treatment. Curr. Opin. Otolaryngol. Head Neck Surg..

[4] Obid R., Redlich M., Tomeh C. (2018). The Treatment of Laryngeal Cancer. Oral Maxillofac. Surg. Clin. North Am..

[5] Levendoski E.E., Leydon C., Thibeault S.L. (2014). Vocal fold epithelial barrier in health and injury: A research review. J. Speech Lang. Hearth Res..

[6] Kim D.H., Kim Y., Kim S.W., Hwang S.H. (2020). Use of narrowband imaging for the diagnosis and screening of laryngeal cancer: A systematic review and meta-analysis. Head Neck.

[7] Alonso-Coello P., Rigau D., Sanabria A.J., Plaza V., Miravitlles M., Martinez L. (2013). Quality and strength: The GRADE system for formulating recommendations in clinical practice guidelines. Arch. Bronconeumol..

[8] Krausert C.R., Olszewski A.E., Taylor L.N., McMurray J.S., Dailey S.H., Jiang J.J. (2011). Mucosal wave measurement and visualization techniques. J. Voice.

[9] Puxeddu R., Sionis S., Gerosa C., Carta F. (2015). Enhanced contact endoscopy for the detection of neoangiogenesis in tumors of the larynx and hypopharynx. Laryngoscope.

[10] Eckel H.E., Simo R., Quer M., Odell E., Paleri V., Klussmann J.P., Remacle M., Sjögren E., Piazza C. (2021). European Laryngological Society position paper on laryngeal dysplasia Part II: Diagnosis, treatment, and follow-up. Eur. Arch. Otorhinolaryngol..

[11] Stanikova L., Walderova R., Jancatova D., Formanek M., Zelenik K., Kominek P. (2018). Comparison of narrow band imaging and the Storz Professional Image Enhancement System for detection of laryngeal and hypopharyngeal pathologies. Eur. Arch. Otorhinolaryngol..

[12] Bergström L.W.E., Finizia C. (2016). The impact of laryngeal biopsy on voice outcomes: A pilot study. Otorhinolaryngol. Head Neck Surg..

[13] Zurek M., Rzepakowska A., Osuch-Wojcikiewicz E., Niemczyk K. (2019). Learning curve for endoscopic evaluation of vocal folds lesions with narrow band imaging. Braz. J. Otorhinolaryngol..

[14] Kaul V., Enslin S., Gross S.A. (2020). History of artificial intelligence in medicine. Gastrointest Endosc.

[15] Repici A., Badalamenti M., Maselli R., Correale L., Radaelli F., Rondonotti E., Ferrara E., Spadaccini M., Alkandari A., Fugazza A. (2020). Efficacy of Real-Time Computer-Aided Detection of Colorectal Neoplasia in a Randomized Trial. Gastroenterology.

[16] Page M.J., McKenzie J.E., Bossuyt P.M., Boutron I., Hoffmann T.C., Mulrow C.D., Shamseer L., Tetzlaff J.M., Akl E.A., Brennan S.E. (2021). The PRISMA 2020 statement: An updated guideline for reporting systematic reviews. BMJ.

[17] Methley A.M., Campbell S., Chew-Graham C., McNally R., Cheraghi-Sohi S. (2014). PICO, PICOS and SPIDER: A comparison study of specificity and sensitivity in three search tools for qualitative systematic reviews. BMC Health Serv. Res..

[18] Whiting P.F., Rutjes A.W., Westwood M.E., Mallett S., Deeks J.J., Reitsma J.B., Leeflang M.M., Sterne J.A., Bossuyt P.M., QUADAS-2 Group (2011). QUADAS-2: A revised tool for the quality assessment of diagnostic accuracy studies. Ann. Intern. Med..

[19] Ni X.G., He S., Xu Z.G., Gao L., Lu N., Yuan Z., Lai S.Q., Zhang Y.M., Yi J.L., Wang X.L. (2011). Endoscopic diagnosis of laryngeal cancer and precancerous lesions by narrow band imaging. J. Laryngol. Otol..

[20] Araújo T., Santos C.P., De Momi E., Moccia S. (2019). Learned and handcrafted features for early-stage laryngeal SCC diagnosis. Med. Biol. Eng. Comput..

[21] Barbalata C., Mattos L.S. (2016). Laryngeal Tumor Detection and Classification in Endoscopic Video. IEEE J. Biomed. Health Inf..

[22] Cho W.K., Lee Y.J., Joo H.A., Jeong I.S., Choi Y., Nam S.Y., Kim S.Y., Choi S.H. (2021). Diagnostic Accuracies of Laryngeal Diseases Using a Convolutional Neural Network-Based Image Classification System. Laryngoscope.

[23] Dunham M.E., Kong K.A., McWhorter A.J., Adkins L.K. (2020). Optical Biopsy: Automated Classification of Airway Endoscopic Findings Using a Convolutional Neural Network. Laryngoscope.

[24] Esmaeili N., Illanes A., Boese A., Davaris N., Arens C., Friebe M. (2019). Novel automated vessel pattern characterization of larynx contact endoscopic video images. Int. J. Comput. Assist. Radiol. Surg..

[25] Inaba A., Hori K., Yoda Y., Ikematsu H., Takano H., Matsuzaki H., Watanabe Y., Takeshita N., Tomioka T., Ishii G. (2020). Artificial intelligence system for detecting superficial laryngopharyngeal cancer with high efficiency of deep learning. Head Neck.

[26] Moccia S., De Momi E., Guarnaschelli M., Savazzi M., Laborai A., Guastini L., Peretti G., Mattos L.S. (2017). Confident texture-based laryngeal tissue classification for early stage diagnosis support. J. Med. Imaging.

[27] Ren J.J., Jing X.P., Wang J., Ren X., Xu Y., Yang Q.Y., Ma L.Z., Sun Y., Xu W., Yang N. (2020). Automatic Recognition of Laryngoscopic Images Using a Deep-Learning Technique. Laryngoscope.

[28] Turkmen H.I., Karsligil M.E., Kocak I. (2015). Classification of laryngeal disorders based on shape and vascular defects of vocal folds. Comput. Biol. Med..

[29] Cho W.K., Choi S.H. (2020). Comparison of Convolutional Neural Network Models for Determination of Vocal Fold Normality in Laryngoscopic Images. J. Voice.

[30] Xiong H., Lin P.L., Yu J.G., Ye J., Xiao L.C., Tao Y., Jiang Z.B., Lin W., Liu M.Y., Xu J.J. (2019). Computer-aided diagnosis of laryngeal cancer via deep learning based on laryngoscopic images. Ebiomedicine.

[31] Davaris N., Voigt-Zimmermann S., Kropf S., Arens C. (2019). Flexible transnasal endoscopy with white light or narrow band imaging for the diagnosis of laryngeal malignancy: Diagnostic value, observer variability and influence of previous laryngeal surgery. Eur. Arch. Otorhinolaryngol..

[32] Zhou H., Zhang J., Guo L., Nie J., Zhu C., Ma X. (2018). The value of narrow band imaging in diagnosis of head and neck cancer: A meta-analysis. Sci. Rep..

[33] Pietruszewska W., Morawska J., Rosiak O., Leduchowska A., Klimza H., Wierzbicka M. (2021). Vocal Fold Leukoplakia: Which of the Classifications of White Light and Narrow Band Imaging Most Accurately Predicts Laryngeal Cancer Transformation? Proposition for a Diagnostic Algorithm. Cancers.

[34] Satankova J., Stanikova L., Svejdova A., Cerny M., Laco J., Chrobok V. (2021). Diagnostic Value of Narrow Band Imaging in Visualization of Pathological Lesions in Larynx and Hypopharynx. Acta Med..

[35] Rzepakowska A., Sielska-Badurek E., Cruz R., Sobol M., Osuch-Wojcikiewicz E., Niemczyk K. (2018). Narrow band imaging versus laryngovideostroboscopy in precancerous and malignant vocal fold lesions. Head Neck.

[36] Zwakenberg M.A., Halmos G.B., Wedman J., van der Laan B., Plaat B.E.C. (2021). Evaluating Laryngopharyngeal Tumor Extension Using Narrow Band Imaging Versus Conventional White Light Imaging. Laryngoscope.

[37] Popek B., Bojanowska-Pozniak K., Tomasik B., Fendler W., Jeruzal-Swiatecka J., Pietruszewska W. (2019). Clinical experience of narrow band imaging (NBI) usage in diagnosis of laryngeal lesions. Otolaryngol. Pol..

[38] Nogues-Sabate A., Aviles-Jurado F.X., Ruiz-Sevilla L., Lehrer E., Santamaria-Gadea A., Valls-Mateus M., Vilaseca I. (2018). Intra and interobserver agreement of narrow band imaging for the detection of head and neck tumors. Eur. Arch. Otorhinolaryngol..

[39] Schünemann H., Brożek J., Guyatt G., Oxman A., GRADE Handbook for Grading Quality of Evidence and Strength of Recommendations (2013). Updated October 2013. The GRADE Working Group. https://guidelinedevelopment.org/handbook.

