# Supplementary material for: Artificial Intelligence in Laryngeal Endoscopy: Systematic Review and Meta-Analysis
Source: J Clin Med. 2022 May 12;11(10):2752. doi: 10.3390/jcm11102752 (PMC9144710; doi:10.3390/jcm11102752)
Supplement: Supplementary file 1 [file jcm-11-02752-s001.zip › Supplementary Table S1.pdf]

Table S1. Literature search strategy

**Search terms**	neural network<br>OR<br>machine learning<br>OR<br>artificial intelligence	AND	larynx<br>OR<br>laryngeal<br>OR<br>vocal fold	AND	lesion<br>OR<br>benign<br>OR<br>malignant<br>OR<br>cancer<br>OR<br>carcinoma
**Databases searched**	PubMed, Embase, Cochrane, Scopus, Web of Science				
**Part of journals searched**	Keywords in all parts of the articles (title, abstract and manuscript)				
**Years of search**	All available till 15.10.2021				
**Language**	English				
**Types of studies to be included**	Qualitative studies				
**Inclusion criteria**	1. Any clinical trial evaluating the application of neural network in endoscopic diagnosis of vocal fold lesions<br>2. The study concerns ENT patients with laryngeal lesions<br>3. The study evaluates at least one of the rates of neural network: accuracy, sensitive, specificity, true positive rate, false positive rate<br>4. No restriction regarding country, patient age, race, gender, publication language, and date				
**Exclusion criteria**	1. Study of neural-network in non-human subjects<br>2. Studies without histopathological diagnosis confirming the benign or malignant nature of the lesions<br>3. Study with data not reliably extracted, duplicate, or overlapping<br>4. Abstract-only papers as preceding papers, conference, editorial, and author response theses and books<br>5. Articles without available full text available<br>6. Case reports, case series, and systematic review studies				
