# Supplementary material for: Artificial Intelligence in Laryngeal Endoscopy: Systematic Review and Meta-Analysis
Source: J Clin Med. 2022 May 12;11(10):2752. doi: 10.3390/jcm11102752 (PMC9144710; doi:10.3390/jcm11102752)
Supplement: Supplementary file 1 [file jcm-11-02752-s001.zip › Supplementary Table S2.pdf]

Table S2. The tailored QUADAS questions.

DOMAIN 1: PATIENT SELECTION
1. Was a consecutive or random sample of patients enrolled?<br>2. Was a case-control design avoided?<br>3. Did the study avoid inappropriate exclusions?<br>4. Has the classification been subjected to various lesions and not just a specific group, e.g. neoplastic changes and fragments of images?<br>5. Were images of different dimensions or whole images of the vocal folds used, and not just standardized image fragments of the same dimensions (which is associated with a lower utility of the network in clinical work)?
**DOMAIN 2: INDEX TEST(S)**
1. Were the index test results interpreted without knowledge of the results of the reference standard?<br>2. If a threshold was used, was it pre-specified?<br>3. Are the results clinically useful or was the aim of the study to improve the web from other articles with no relevance to its clinical utility?<br>4. Are there any cross-tables of the results of the artificial intelligence and not only the values of the rates (accuracy, sensitivity, specificity etc.)?<br>5. Was only the validation of the artificial intelligence performed without testing on another or additional set of images?
**DOMAIN 3: REFERENCE STANDARD**
1. Is the reference standard likely to correctly classify the target condition?<br>2. Were the reference standard results interpreted without knowledge of the results of the index test?<br>3. Was the diagnosis confirmed by histopathological examination when classifying malignant lesions?<br>4. Has the diagnosis been made by an ENT specialist with appropriate experience when classifying benign lesions, e.g. nodules, polyps?
**DOMAIN 4: FLOW AND TIMING**
1. Was there an appropriate interval between index test(s) and reference standard?<br>2. Did all patients receive a reference standard?<br>3. Did patients receive the same reference standard?<br>4. Were all patients included in the analysis?<br>5. Were all images classified in the same way (diagnosis of ENT specialist or histopathological examination)?<br>6. Were all images previously classified by the same ENT or histopathological examination?<br>7. Were all available images used without any exclusions?
