# Supplementary material for: Artificial Intelligence in Laryngeal Endoscopy: Systematic Review and Meta-Analysis
Source: J Clin Med. 2022 May 12;11(10):2752. doi: 10.3390/jcm11102752 (PMC9144710; doi:10.3390/jcm11102752)
Supplement: Supplementary file 1 [file jcm-11-02752-s001.zip › Supplementary Table S3.pdf]

Table S3. Summary table of the studies included in the meta-analysis.

Author	Year	Study design	No. Patients	No. Images (total)	Aims and outcomes	Preprocessing and methodology	CNN	Reference standard	Whole images	NBI	Terminology in article	Corresponding vascular pattern in Ni classification	No. Patients in the class	No. Images in the class
Ara ´ujo	2019	Retrospective	33	1320	Classification into four tissue classes: He (healthy tissue), Hbv (tissue with hypertrophic vessels), Le (tissue with leukoplakia) and IPCL (tissue with intrapapillary capillary loops)	Gaussian smoothing, texture-based global descriptors, first-order statistics and CNN-based learned features	1	Histopathology and ENT diagnose	0	1	He (healthy tissue)	I	-	330
											Hbv (tissue with hypertrophic vessels)	II	-	330
											Le (tissue with leukoplakia)	III	-	330
											IPCL (tissue with intrapapillary capillary loops)	IV + V	-	330
Barbalata	2016	Retrospective	60	120	Classification into malignant versus benign lesions	Specular Reflections Removal, ROI detection, Blood Vessel Extraction, Classification based on vessel size	0	Histopathology	1	1	Benign lesions	I + II	17	34
											Malignant lesions	IV + V	43	86
Cho	2021	Retrospective	4106	4106	Classification into normal larynx, cysts, nodules, polyps, leukoplakia, laryngeal papillomas, Reinke's edema, granulomas and vocal cord palsies	Manually cropping vocal fold images to choose ROI, CNN with pretrained model	1	Histopathology and ENT diagnose	1	0	normal larynx	I	742	742
											cysts	I + II	347	347
											nodules	I	171	171
											polyps	I + II	948	948
											leukoplakia	III	775	775
											laryngeal papillomas	IV+V	120	120
											Reinke's edema	I + II	149	149
											granulomas	I + II	296	296
											vocal cord palsies	I	558	558
Dunham	2020	Retrospective		19353	Binary classification into malignant–premalignant and benign lesions	Selection using a structural similarity index (SSIM), image data augmentation, CNN with pretrained model	1	Histopathology and ENT diagnose	1	0	**benign class**	I + II	-	17423
											normals tissue	I	-	3444
											nodules	I	-	1816
											papillomas	IV + Va	-	3633
											polyps	I + II	-	4577

					Classification of benign lesions: normal, nodules, papilloma, polyps and webs			Histopathology and ENT diagnose			webs	I	-	3953
											**malignant–premalignant class**	III + IV + V	-	1930
											leukoplakia	III	-	925
											carcinoma	IV+V	-	1005
Esmaeili	2019	Retrospective	32	1485	Classification of vascular patterns: “order”, “disorder” and “very disorder”	Homogenization of images, vessel enhancement with Frangi filter, skeletonization procedure of vessels, calculating five indicators (HGD, RIA, ANG, DIS, CUR) of vascular patterns, classification using SVM, k-nearest neighbors (kNN), and random forests (RF)	0	Histopathology and ENT diagnose	0	1	order vascular pattern	I	32	1485
											disorder vascular pattern	II		
											very disorder vascular pattern	IV+V		
			20	890	Classification of benign histopathologies - cyst, polyp & reinke’s edema, papilloma, and dysplasia mild			Histopathology			**benign class**	I + II	20	890
											cyst	I + II	3	150
											polyp	I + II	4	130
											reinke’s edema	I + II	5	250
											papilloma	IV + Va	5	230
											dysplasia mild	IV	3	130
			11	465	Classification of malignant histopathologies: dysplasia severe, carcinoma in situ and carcinoma			Histopathology			**malignant histopathologies**	IV + V	11	465
											dysplasia severe	IV + V	4	130
											carcinoma in situ	IV + V	4	155
											carcinoma	V	3	180
			31	1355	Classification into benign and malignant histopathologies			Histopathology			-	-	31	1355
Inaba	2020	Retrospective	374	2400	Classification into superficial laryngopharyngeal cancer and normal tissue	Manually cropping vocal fold images to choose ROI, CNN with pretrained model	1	Histopathology	1	1	superficial laryngopharyngeal cancer (SLPC)	IV + V	174	800
											normal laryngopharyngeal mucosa	I	200	1600
Moccia	2017	Retrospective	33	1320	Classification into: tissue with IPCL-like vessels, leukoplakia, tissue with hypertrophic	Anisotropic diffusion filtering, specular reflections masking, selecting of squared patches, extraction of	0	Histopathology and ENT diagnose	0	1	healthy tissue	I	-	330
											tissue with hypertrophic vessels	II	-	330
											leukoplakia	III	-	330

					vessels and healthy tissue	texture-based global descriptors and first-order statistics, classification using SVM, k-nearest neighbors (kNN), naive Bayes (NB) and random forest (RF)					tissue with IPCL-like vessels	IV + V	-	330
Ren	2020	Retrospective	9231	24667	Classification into normal tissue, vocal nodule, polyps, leukoplakia and malignancy	Removing duplicated images, with low resolution, and without vocal cords, classification using CNN with pretrained model	1	Histopathology and ENT diagnose	1	0	normal tissue	I	-	10215
											vocal nodule	I	-	5807
											polyps	I+II	-	2995
											leukoplakia	III	-	2120
											malignancy	IV+V	-	3530
Turkmen	2015	Retrospective	70	124	Categorization into vocal folds into healthy, nodule, polyp, sulcus vocalis, and laryngitis classes	Detection of vocal folds based on Histogram of Oriented Gradients (HOG), segmentation of glottis, and normalization of vocal fold images; extraction of vocal fold edge and vessel features; classification using Naive Bayes, multilayer perceptron, k-nearest neighbors (KNN), support vector machine (SVM), and random forest (RF)	0	ENT diagnose	0	0	healthy tissue	I	14	28
											nodule	I	10	20
											polyp	I + II	16	17
											sulcus vocalis	I	15	29
											laryngitis	II	15	30
Xiong	2019	Retrospective	2208	14897	Classification into Urgent versus Non-urgent subjects	Manually selecting images, CNN with pretrained model	1	Histopathology	1	0	**Non-urgent subjects**	I + II	1712	10554
											**Urgent subjects**	IV + V	496	4388
					Classification into laryngeal cancer						normal tissues (NORM)	I	770	3602

					(LCA), precancerous laryngeal lesions (PRELCA), benign laryngeal tumors (BLT) and normal tissues (NORM)						benign laryngeal tumors (BLT)	I+II	942	6952
											precancerous laryngeal lesions (PRELCA)	III + IV + V	246	1936
											laryngeal cancer (LCA)	V	250	2452
Cho	in press	Retrospective	2216	2216	Classification into normal and abnormal vocal fold tissue	Manually cropping vocal fold images to choose ROI, CNN with pretrained model	1	Histopathology	1	0	normal tissue	I	899	899
											abnormal tissue	II + III + IV+ V	1317	1317
