# Supplementary material for: Artificial Intelligence in Laryngeal Endoscopy: Systematic Review and Meta-Analysis
Source: J Clin Med. 2022 May 12;11(10):2752. doi: 10.3390/jcm11102752 (PMC9144710; doi:10.3390/jcm11102752)
Supplement: Supplementary file 1 [file jcm-11-02752-s001.zip › Supplementary Table S4.pdf]

Table S4. The results of the QUADAS-2 bias and applicability evaluation.

Study (Title/Author/Year)			RISK OF BIAS				APPLICABILITY CONCERNS		
			PATIENT SELECTION	INDEX TEST	REFERENCE STANDARD	FLOW AND TIMING	PATIENT SELECTION	INDEX TEST	REFERENCE STANDARD
Learned and handcrafted features for early-stage laryngeal SCC diagnosis	Ara ´ujo	2019	☹	?	☺	☺	☺	☺	☺
Optical Biopsy: Automated Classification of Airway Endoscopic Findings Using a Convolutional Neural Network	Dunham	2020	☺	☺	☺	☺	☺	☺	☺
Novel automated vessel pattern characterization of larynx contact endoscopic video images	Esmaeili	2019	?	☺	☺	☺	☺	☺	☺
Artificial intelligence system for detecting superficial laryngopharyngeal cancer with high efficiency of deep learning	Inaba	2020	☺	☺	☺	☺	☺	☺	☺
Confident texture-based laryngeal tissue classification for early stage diagnosis support	Moccia	2017	☹	☺	☺	☺	☺	☺	☺
Automatic Recognition of Laryngoscopic Images Using a Deep-Learning Technique	Ren	2020	☺	☺	☺	☺	☺	☺	☺
Classification of laryngeal disorders based on shape and vascular defects of vocal folds	Turkmen	2015	?	☹	?	☺	☺	☺	☺
Computer-aided diagnosis of laryngeal cancer via deep learning based on laryngoscopic images	Xiong	2019	☺	☺	☺	☺	☺	☺	☺
Diagnostic Accuracies of Laryngeal Diseases Using a Convolutional Neural Network-Based Image Classification System	Cho	2021	☺	☺	☺	☺	☺	☺	☺
Laryngeal Tumor Detection and Classification in Endoscopic Video	Barbalata	2016	☺	?	☺	☺	☺	☺	☺
Comparison of Convolutional Neural Network Models for Determination of Vocal Fold Normality in Laryngoscopic Images	Cho	in press	☺	?	☺	☺	☺	☺	☺
