# Supplementary material for: Artificial Intelligence in Laryngeal Endoscopy: Systematic Review and Meta-Analysis
Source: J Clin Med. 2022 May 12;11(10):2752. doi: 10.3390/jcm11102752 (PMC9144710; doi:10.3390/jcm11102752)
Supplement: Supplementary file 1 [file jcm-11-02752-s001.zip › Supplementary Table S5.pdf]

Table S5. Raw data of the included studies.

TP – true positive; TN – true negative; FP – false positive; FN – false negative; SE – standard error; LCI – lower 95% confidence interval; UCI – upper 95% confidence interval

Author	Year	TP	TN	FP	FN	ACCURACY	SENSITIVITY				SPECIFICITY			
							Value	SE	95% LCI	95% UCI	Value	SE	95% LCI	95% UCI
**IDENTIFICATION OF HEALTHY LARYNGEAL TISSUE**														
Dunham	2020	40	190	10	10	0.92	0.80	0.06	0.69	0.91	0.95	0.02	0.92	0.98
Moccia	2017	323	971	19	7	0.98	0.98	0.01	0.96	0.99	0.98	0.00	0.97	0.99
Ren	2020	100	395	5	0	0.99	1.00	0.00	1.00	1.00	0.99	0.01	0.98	1.00
Turkmen	2015	24	94	2	4	0.95	0.86	0.07	0.73	0.99	0.98	0.01	0.95	1.01
Xiong	2019	528	2,075	94	132	0.92	0.80	0.02	0.77	0.83	0.96	0.00	0.95	0.97
Cho	2021	694	3,309	73	30	0.97	0.96	0.01	0.94	0.97	0.98	0.00	0.97	0.98
Cho	in press	894	1,317	0	5	1.00	0.99	0.00	0.99	1.00	1.00	0.00	1.00	1.00
**DIFFERENTIATION BETWEEN BENING AND MALIGNANT LESIONS**														
Dunham	2020	46	47	4	3	0.93	0.94	0.03	0.87	1.01	0.92	0.04	0.85	1.00
Esmaeili	2019	437	764	126	28	0.89	0.94	0.01	0.92	0.96	0.86	0.01	0.84	0.88
Inaba	2020	328	787	13	18	0.97	0.95	0.01	0.92	0.97	0.98	0.00	0.97	0.99
Moccia	2017	284	636	10	36	0.95	0.89	0.02	0.85	0.92	0.98	0.00	0.98	0.99
Ren	2020	90	294	4	7	0.97	0.93	0.03	0.88	0.98	0.99	0.01	0.97	1.00
Xiong	2019	188	867	73	48	0.90	0.80	0.03	0.75	0.85	0.92	0.01	0.91	0.94
